# Self image, stress level and quality of life in adolescents patients with idiopathic scoliosis

**DOI:** 10.1186/1748-7161-8-S1-O57

**Published:** 2013-06-03

**Authors:** E Kinel, T Kotwicki, A Podolska, M Białek, W Stryła

**Affiliations:** 1 Department of Rehabilitation, University of Medical Sciences, Poznan, Poland; 2 Department of Paediatric Orthopaedics and Traumatology, University of Medical Sciences, Poznan, Poland; 3 FITS Center, Jawor, Poland

## Background

Adolescent idiopathic scoliosis (AIS) is a complex and progressive condition, which can affect a patient’s quality of life (QoL).

## Aim

The aim of the study was to evaluate the self-image, the stress level, and the quality of life in adolescents with idiopathic scoliosis, who are under brace treatment.

## Methods

It involved 59 adolescents, (50 girls, 9 boys), ages ranging between 10.0 and 17.0 years, all with Adolescent Idiopathic Scoliosis (AIS) with Cobb angle between 20-58 degrees. The adolescents were wearing the same kind of brace (Chêneau orthosis) for more than 6 months, for at least 16h per day. Three questionnaires were used: Trunk Appearance Perception Scale (TAPS), Bad Sobernheim Stress Questionnaire (BSSQ Brace, BSSQ Deformity), and Brace Questionnaire (BrQ).The TAPS is a tool to evaluate subjective impression of trunk deformity, which includes 3 sets of figures that depict the trunk, from 3 viewpoints: looking toward the back, looking toward the head with the patient bending over, and looking toward the front. BSSQ is the questionnaire that estimates the stress scoliosis patients have, whilst wearing their brace. The BrQ is an instrument for measuring quality of life of scoliotic adolescents, who are being treated conservatively, with wearing of a corrective brace. The analysis considered the type of treatment, curve location, correlation of the total scores with age, Cobb angle and Bunnell rotation angle.

## Results

The age was 14.1 ± 1.7 years. Cobb angle was 33.2 ± 9.5 degrees. The TAPS median for the total score was 4. Adolescents revealed higher score with BSSQ Deformity (median = 14) comparing to BSSQ Brace (median = 11). The mean score for BrQ was 76.6 ± 12.1.

## Conclusion

The adolescents who were under brace treatment suffered moderate level of stress from the deformity. Conservative treatment did not severely impact on the quality of life of scoliotic adolescents.
